# Monolithic-Structured Single-Layered Textile-Based Dye-Sensitized Solar Cells

**DOI:** 10.1038/srep34249

**Published:** 2016-10-06

**Authors:** Min Ju Yun, Seung I. Cha, Han Seong Kim, Seon Hee Seo, Dong Y. Lee

**Affiliations:** 1 Nano Hybrid Research Center, Creative and Fundamental Research Division, Korea Electrotechnology Research Institute, 12, Bulmosan-ro 10beon-gil, Seongsan-gu, Changwon, 51543, Republic of Korea; 2 Department of Organic Material Science and Engineering, Pusan National University, 2, Busandaehak-ro 63beon-gil, Geumjeong-gu, Busan, 46241, Republic of Korea; 3 Department of Electro-functionality Materials Engineering, University of Science and Technology, 12, Bulmosan-ro 10beon-gil, Seongsan-gu, Changwon, 51543, Republic of Korea.

## Abstract

Textile-structured solar cells are frequently discussed in the literature due to their prospective applications in wearable devices and in building integrated solar cells that utilize their flexibility, mechanical robustness, and aesthetic appearance, but the current approaches for textile-based solar cells—including the preparation of fibre-type solar cells woven into textiles—face several difficulties from high friction and tension during the weaving process. This study proposes a new structural concept and fabrication process for monolithic-structured textile-based dye-sensitized solar cells that are fabricated by a process similar to the cloth-making process, including the preparation of wires and yarns that are woven for use in textiles, printed, dyed, and packaged. The fabricated single-layered textile-based dye-sensitized solar cells successfully act as solar cells in our study, even under bending conditions. By controlling the inter-weft spacing and the number of Ti wires for the photoelectrode conductor, we have found that the performance of this type of dye-sensitized solar cell was notably affected by the spacing between photoelectrodes and counter-electrodes, the exposed areas of Ti wires to photoelectrodes, and photoelectrodes’ surface morphology. We believe that this study provides a process and concept for improved textile-based solar cells that can form the basis for further research.

Wearable electronic devices have been the subject of much attention and development in recent years, along with subsequent demands for portable electrical sources to be integrated into small devices. In addition, solar cells are expected to be a promising energy-harvesting source on buildings’ interior walls in the near future. From this point of view, among the various currently available solar-power sources, dye-sensitized solar cells (DSSCs) have several advantages, including low production costs, high energy-conversion efficiency (even under weak illumination conditions), and an aesthetically pleasing appearance^1^,^2^. In order to find applications for wearable devices as solar-power sources or to build integrated energy-harvesting sources, the material must be lightweight, highly flexible, affordable, and aesthetically appealing. The transparent oxide-coated (TCO) plastic substrate-based flexible solar cells that are currently used have certain limitations (especially in their mechanical robustness) due to the fragile nature of TCO films. As a result, TCO-free dye-sensitized solar cells have garnered much attention in recent years as a possible replacement^3^,^4^,^5^,^6^,^7^,^8^,^9^,^10^.

As a wearable concept for meeting future individual needs to incorporate solar cells into clothing, backpacks, and other belongings, and as a building-integrated or interior-design concept, textile structures are highly suitable materials; many researchers have worked on developing textile-structured DSSCs as a result. In addition, textile structures have distinctive mechanical robustness under highly deformed conditions, including bending, rolling, and even folding. Textile-based dye-sensitized solar cells are thus a promising candidate for next-generation urban energy-harvesting devices. The general approach to obtaining textile photovoltaic forms starts with integrating components within the fibre or wire. In this approach, it is generally believed that the solar-cell fibres or wires can be easily woven into textiles. During the actual weaving process, however, the fibres undergo severe friction and tension, which makes it difficult to weave dense textiles (such as is the case with most ‘real’ fabrics), and the electrode layer may suffer damage from the friction. Other difficulties are that the electrodes (which consist of different fibre types) are limited in length, and the process involved in manufacturing continuous fabrics presents various problems^11^,^12^,^13^,^14^,^15^,^16^,^17^.

In order to avoid these problems, we have proposed a novel two-type textile-structured DSSC. In one method, each electrode is deposited on each textile type’s substrate; a heat-treatment process is then applied at each proper temperature; next, the spacer cloth is placed between each electrode-deposited substrate and is attached by a sewing method to assemble the cell^18^. In the second method, each electrode is deposited and heat-treated before insertion into the textile, where it will act as the weft in the weaving process that creates the textile-type DSSCs^19^. When performing these types of DSSCs, however, the preparation of electrodes and the assembly processes are separate, so it is impossible to perform the process continuously. To solve this problem, we first fabricated the core-integrated cell^9^ before depositing the electrodes to achieve a continuous process.

In this study, we have proposed new fabric types for DSSCs using wires and glass-fibre yarn for the warp and weft to weave materials into a single layer, which is similar to the process for creating real textiles. In this process, photoelectrodes, counter-electrodes, and glass fibre (which is used to fill the electrolytes and prevent electrical shorts), are woven into a monolithic-structured single-layered textile in which the electrodes are arranged side by side to form the warp. The use of this process and structure allows for densely and continuously fabricating materials for as long as is desired—much like real fabric—and thus can be applied to wearable devices or for integration into fabric applications. Deposition of the electrode paste was also conducted after the fabrication of the textile-structure cell to prevent destruction of the electrode during the weaving process.

## Results and Discussion

**Figure 1:**
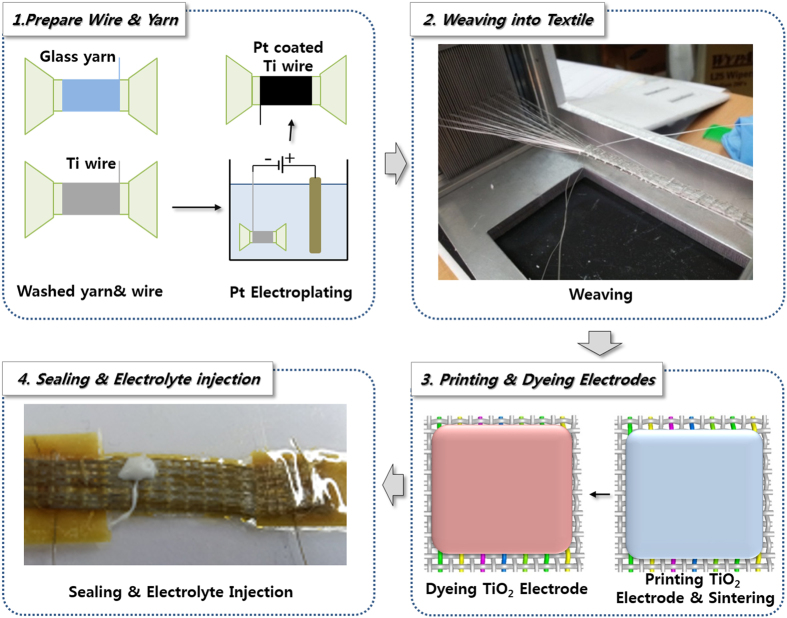
Schematic illustration of the fabrication process for monolithic-structured single-layered textile-based dye-sensitized solar cells, consisting of four steps: (1) preparation of wires and yarns, (2) weaving into textiles, (3) printing and dyeing, and (4) packaging.

In this study, monolithic-structured single-layer textile DSSCs were fabricated using the concept of the core-integrated structure^9^, which is used for alternative solutions to the problems discussed above; such materials are TCO-free and have the flexible characteristics of a typical sandwich structure (Fig. S1). The fabrication process for monolithic-structured dye-sensitized solar cells based on a single layer of textile consists of four main steps, as illustrated in Fig. 1. In the first step, the wires and yarns that will be woven into a textile are prepared. The 100-μm-diameter Ti metal wires were utilized for the conducting wires for the photoelectrodes and counter-electrodes, while glass-fibre yarns were used as the spacers between electrodes to avoid electrical shorts and to maintain their electrolytes. The wires and yarns were cleaned via sonication in water, ethanol, and acetone, followed by drying to remove any contamination stains during the fabrication of wires and yarns. For the Ti wires that were utilized for the counter-electrodes, the Pt was electro-deposited before the weaving process. As a result, three kinds of wires and yarns were prepared for the weaving process, including Ti wires (photoanode conductors), Pt-coated Ti wires (counter-electrode catalysts and conductors), and glass-fibre yarns (spacers).

In the second step, the wires and yarns were woven into a single textile layer in which the Ti wires and Pt-coated Ti wires (as counter-electrodes and glass-fibre yarns, respectively) were aligned in the warp direction; the glass-fibre yarns interlocked with the wires to form weft yarns in a table-top loom, as shown in Fig. 1. Detailed photographs of the aligned wires and glass-fibre yarns as the warp, woven into a single-layer textile, as well as fabrication of the monolithic single-layer textile cells, are shown in Fig. S2.

In the third step, the TiO_2_ nanoparticle paste was printed on the woven textile and sintered to form photoanodes; the dye was then stained into the photoanodes by immersing the photoanode-coated textile in the dye solution. The photoanode-prepared textile was then sealed by transparent and flexible films by hot pressing or hot rolling; electrolytes were then injected into the textile as the fourth and final step (Fig. S2).

This four-step process produces a metal wire/glass fibre-based textile that the photoanode is then deposited upon, as shown in Fig. 1. It should be emphasized that this suggested fabrication process is far different from the conventional dye-sensitized solar-cell fabrication process. Our suggested process is based essentially on the traditional textile-making process (including preparing yarns and weaving), which enables continuous fabrication for as long as is desired; this is similar to creating real fabric using a roll from the front part of a table-top loom (Fig. S3) for printing, dyeing, and packaging. We therefore expect that if proper equipment is employed, the continuous textile process could be applied to the solar-cell fabrication process.

**Figure 2:**
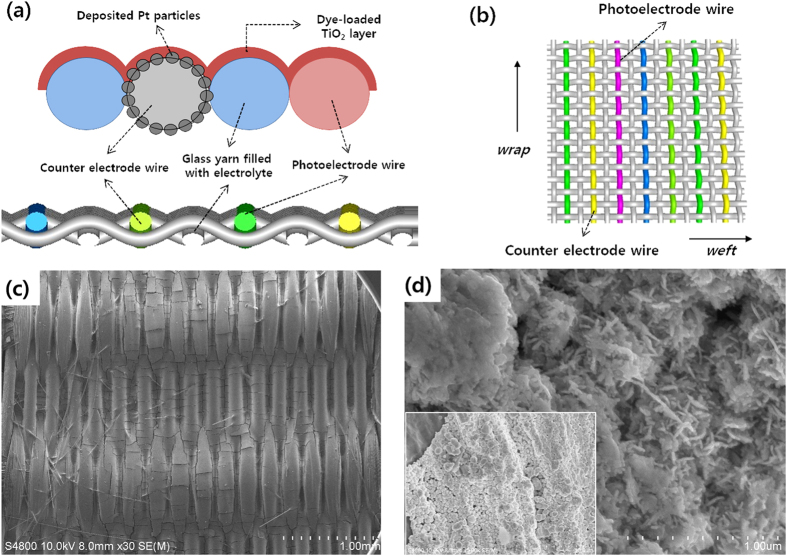
Schematic illustration of (**a**) cross-sectional view and (**b**) planar view of the monolithic-structured single textile based DSSCs. (**c**) Scanning electron microscopy (SEM) micrograph of the surface of TiO_2_ photoelectrode deposited on the prepared single-layered textile of Ti wire, Pt-coated Ti wire, and glass-fibre yarns. (**d**) SEM micrograph showing the coated Pt on the Ti wires for the counter-electrodes; the inset shows a low-magnification micrograph displaying the uniform coverage of Pt on the Ti wires.

The detailed structure of the fabricated dye-sensitized solar cells based on a single textile layer is illustrated in Fig. 2(a). The Ti wire, glass-fibre yarn, and Pt-coated Ti wires are located alongside one another, as shown in the cross-sectional illustration in Fig. 2(a); the photoelectrode, consisting of TiO_2_ nanoporous film loaded with dye, is then deposited on these materials. The glass-fibre yarn weft is interwoven with these wires and yarns to form a textile. The inserted glass-fibre yarn between the Pt-coated Ti wires, which acts as the counter-electrode, prevents electrical shorts between electrodes and behaves as an electrolyte reservoir after electrolyte injection. The coated photoelectrodes for the most part resemble the structures of monolithic dye-sensitized solar cells, except that the underlying structure consists of textiles^10^,^17^,^20^.

The planar view of this structure shown in Fig. 2(b) provides a clearer picture of the monolithic-structured dye-sensitized solar cell based on textile structures. The lead that projects from the external circuit from the solar cell forms a possible extension of the counter-electrode wires and Ti wires to the outside of the sealant. The TiO_2_ photoelectrode deposited on the monolithic-structured single-layer textile showed uniformity according to the surface profile of the textile, as shown in Fig. 2(c). We may therefore expect that the modification of the weaving patterns in the underlying textile will also change the morphology of the photoelectrodes^21^. The surfaces of the Pt-coated Ti wire, which act as counter-electrodes, are shown in Fig. 2(d). The nanostructured Pt was deposited on the Ti wire surface with the morphology of a vertical assembly of nano-flakes via electro-plating. This structure uniformly covered the Ti wire, as shown in Fig. 2(d). Further analysis of the surface structure of the Ti wire and Pt-deposited Ti wire was conducted using X-ray powder diffraction (XRD) characterization. The Pt phases at 40.0°, 46.6°, and 67.9° were present in the XRD graph; the broad peaks were due to the nano-scale size of the particles (Fig. S4).

**Figure 3:**
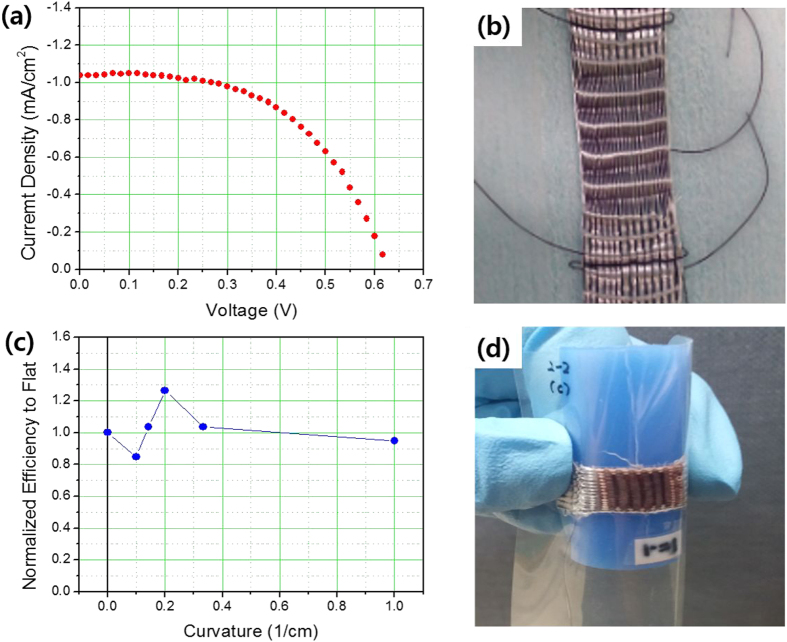
(**a**) The relationship between current density and voltage for the monolithic-structured single-layered textile-based dye-sensitized solar cells, woven with the spacing of wefts at 0.75 mm under 1 sun, 1.5 AM condition. (**b**) Photograph of underlying single-layer textiles before the deposition of TiO_2_ photoelectrodes. (**c**) Plot showing variations of normalized energy-conversion efficiency according to bending curvature (1/radius of curvature) to that measured in a flat sample. (**d**) Photograph of monolithic-structured single-layered textile-based DSSCs bending around a rod of 1 cm radius curvature.

The process and structure of the textile-based monolithic-structured dye-sensitized solar cells are shown in Figs 1 and 2, respectively; these cells convert light to electricity. Figure 3(a) shows the current density and voltage relationships. The short-circuit current density is measured as 1.04 mA cm^−2^, the open-circuit voltage as 0.62 V, and the fill factor as 0.54. In this case, the single Ti wire was used for the conductor of the photoanodes, and the single Pt-coated Ti wire was used for the counter-electrode. The spacing between the wefts (i.e. inter-weft spacing) was maintained at 0.75 mm during the weaving process, as show in Fig. 3(b), which is the underlying textile structure that the photoanode was deposited upon. Although the basic performance was not yet sufficient for regular performance, because it was not yet optimized for performance, it was very impressive that the bending of this cell did not degrade the performance until the radius of the curvature reached 1 cm, as shown in Fig. 3(c,d). This suggests that the textile-structured dye-sensitized solar cells would be quite useful for flexible solar cells when they are prepared via weaving first, followed by a coating of electrodes, as shown in Fig. 1.

**Figure 4:**
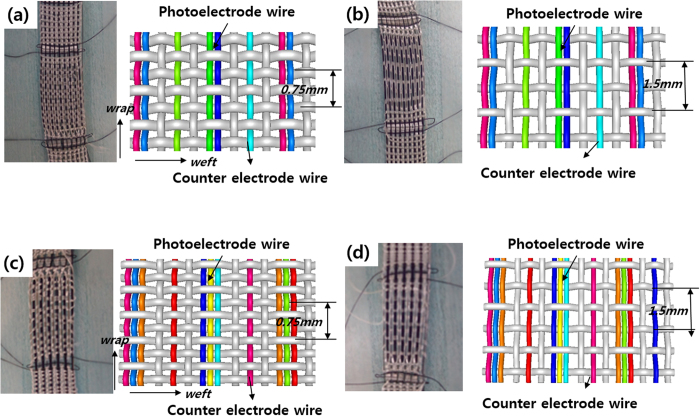
(**a**) Photograph and schematic illustration of underlying textiles for the sample with inter-weft spacing of 0.75 mm; two Ti wires were employed for the photoelectrode. (**b**) Underlying textiles with inter-weft spacing of 1.5 mm; two Ti wires were employed. (**c**) Underlying textiles with inter-weft spacing of 0.75 mm; three Ti wires were employed. (**d**) Underlying textiles with inter-weft-spacing of 1.5 mm; three Ti wires were employed.

In order to improve the energy-conversion efficiency of the monolithic-structured fabric-type DSSCs, we increased the number of Ti wires from single wires to two or three basket-type wires (i.e. they were woven together as the weft), as shown in Fig. 4; this weft acted as the substrate for the photoanode. We expected that the increase in Ti wires beneath the photoelectrode would enhance electron gathering from the photoelectrode and would reduce recombination loss in the electrodes, which is induced by the long diffusion path from the generation site to the gathering interface. At the same time, we changed the interspacing between the glass-fibre weft interlocking the warp wires and yarns from 0.75 mm to 1.5 mm. The increase in the weft interspacing induced a flatter surface profile of the textile base, which led to smoother photoelectrode morphology. This was because the morphology of the photoelectrode followed the surface profile of the underlying textile, as shown in Fig. 2(c). In addition, the exposed area of the Ti wires for the conduction of the photoelectrode was increased by increasing the weft interspacing, as shown in Fig. 4(a–d).

**Figure 5:**
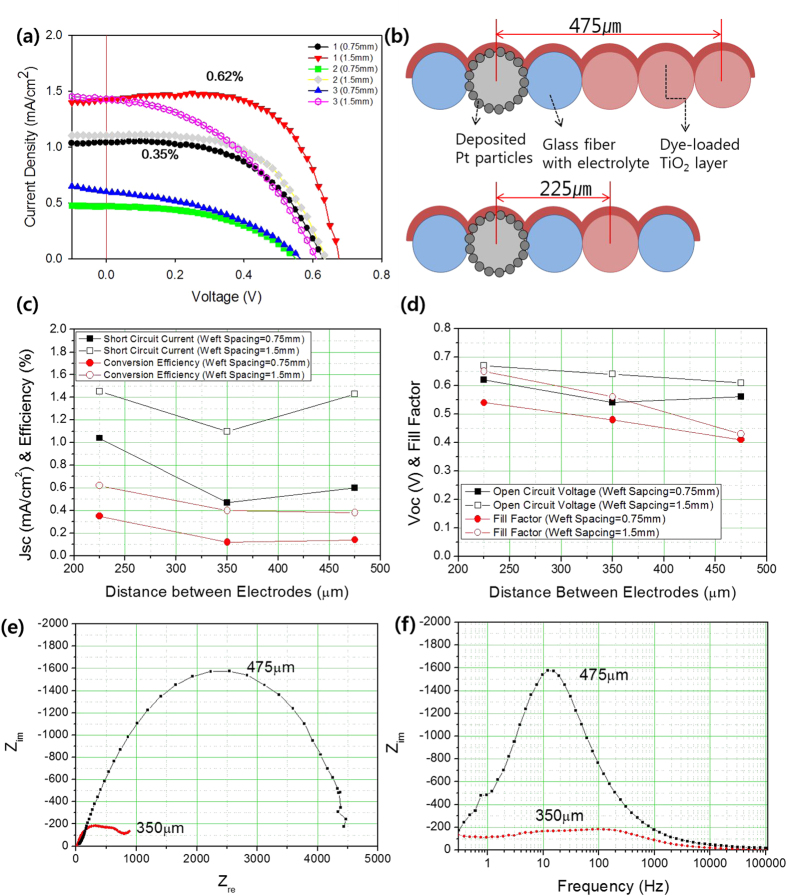
(**a**) Relationship between current density and voltage of monolithic-structured single-layered textile-based dye-sensitized solar cells according to the number of wires for photoelectrodes and inter-weft spacing. (**b**) Schematic illustration of the indicated distances between the centre of the photoelectrode and the counter-electrode wire by increasing Ti wires for the photoelectrode. The plot indicates (**c**) short-circuit current and energy-conversion efficiency and (**d**) fill factor and open-circuit voltage of monolithic-structured single-layered textile-based dye-sensitized solar cells according to the distance between electrodes and inter-weft spacing. (**e**) Nyquist plot and (**f**) Bode plot for monolithic-structured single-layered textile-based dye-sensitized solar cells under 1 sun, 1.5 AM condition according to distances between electrodes.

The increase in the weft interspacing enhanced the materials’ performance, as shown in Fig. 5(a), but the increased number of Ti wires for the photoelectrode conductor decreased their performance, as shown in Fig. 5(a). Considering the expected effect of the number of Ti wires, which increases the area that is exposed to the photoelectrodes, it could be supposed that other effects become more dominant by increasing the number of Ti wires. To solve this problem, we have proposed the spacing between electrodes shown in Fig. 5(b). The increase in the number of Ti wires beneath the photoelectrode means that the average distance between the photoelectrodes and the counter-electrodes also increases, as illustrated in Fig. 5(b); the plot of the performance of the textile-based monolithic-structured dye-sensitized solar cell according to inter-electrode spacing shown in Fig. 5(c,d) confirms this postulation. This plot shows that the increased inter-electrode spacing decreased the short-circuit current, open-circuit voltage, and fill factor; in particular, the fill factor and short-circuit current decreased dramatically.

We then conducted repeatability tests with three samples under the same conditions and variations in order to achieve convincing results and to demonstrate the feasibility of monolithic-structured single-layer textile DSSCs. The performances of all trial cells are shown in Table S1; the performances are also supported from the data of the electrochemical impedance spectroscopy (EIS) results, shown in Fig. 5(e,f). In the Nyquist plot, the wider inter-electrode spacing induced high impedance when compared with the 35-μm distance between the electrodes (RCT, 600 Ω), while the Bode plot shows that the corresponding frequency was below 100 Hz, which was determined by the diffusion of ions in the electrolytes.

The data also shows that the increasing inter-weft spacing considerably enhanced the performance of the single-layer textile-based monolithic-structured dye-sensitized solar cells. As mentioned earlier, the larger inter-weft spacing induced both a larger exposed area of the Ti wires to the photoelectrodes and a smoother morphology of the photoelectrodes. Although at this stage it is not clear which technique is more effective for improved performance, we are confident that both techniques notably affected the performance of the textile-based dye-sensitized solar cells.

The stability of the monolithic-structured textile DSSCs with a 225-μm distance between the electrodes and 0.75 mm spacing is shown in Fig. S5; the initial energy-conversion efficiency (0.35%) decreased to 0.21% and 0.18% after 5 and 7 days, respectively. Decreased efficiency is related to the evaporation of the liquid electrolytes, which means that the sealing method should be improved; in addition, the liquid electrolytes should be replaced with solid state or quasi-solid state electrolytes to ensure the stability of the textile DSSCs for application purposes.

The modification of the structure of the underlying textile structures and the investigation of the performance according to the variation of these structures provided important design rules for fabricating high-performance textile solar cells. The results suggest that the contact area between the conducting wires or yarns for the conductor and the photoelectrode should be maximized in order to effectively gather the generated electrons to the conducting wires. At the same time, the spacing between the photoelectrodes and the counter-electrodes should be made as short as possible to avoid losses from conduction through the electrolytes. In addition, the surface of the photoanodes should be controlled to more effectively gather the electrons through diffusion of the electrodes. These situations may be obtained by the new invention of textile patterns that will meet all of the suggested conditions; we expect that researchers will invent these patterns within a short time in order to reveal the optimal textile patterns as an expansion of this study. It should be noted, however, that in this study we have proposed a new concept for textile-based solar cells (i.e. single-layer textile-based monolithic-structured dye-sensitized solar cells) that successfully act as solar cells even under bending conditions. In addition, we have suggested the critical parameters that will further improve the performance of textile solar cells; this will also require further investigation. Most importantly, we have proposed a new concept for fabrication processes that resembles the traditional cloth-making process, which consists of the preparation of yarn and wires, weaving, printing and dyeing, and packaging.

In summary, single-layered textile-based monolithic-structured dye-sensitized solar cells were successively fabricated in this study by using concepts from the traditional cloth-making process, including wire and yarn preparation, weaving, printing and dyeing, and packaging; this process utilizes Ti wires, Pt-coated Ti wires, and glass-fibre yarns. The processed dye-sensitized solar cells have maintained their energy-conversion performance under a bending condition of 1 cm radius of curvature. By controlling the inter-weft spacing and the number of Ti wires for the photoelectrode conductor, we have found that the performance of this type of dye-sensitized solar cell is notably affected by the spacing between photoelectrodes and counter-electrodes, the exposed area of the Ti wires to the photoelectrodes, and the surface morphology of the photoelectrodes. We believe that this study has provided a basic process and design concept for improved textile-based solar cells with optimized performance that can be improved by further research in the near future.

## Methods

A 100-μm-diameter Ti wire (iNexus) and glass-fibre yarn (D450 1/2, Hyunmin Fiber) were used as the warp, and glass fibre (D450 1/2, Hyunmin Fiber) was used as the weft. A table-top loom produced by Daesung High-tech was utilized for the weaving process. Before weaving, the wires for the electrodes were rinsed with acetone, ethanol, and DI water by sonication and dried with nitrogen gas. For the counter-electrodes, the wires were electro-plated for Pt deposition. Electro-deposition was performed using an aqueous solution of 50 mM H_2_PtCl_6_∙6H_2_O (Sigma Aldrich) at room temperature with 2 V DC power for 5 min, then calcined at 180 °C for 1 hour.

After weaving the textile cell, TiO_2_ paste containing 20 nm TiO_2_ nanoparticles (EnB Korea) was coated using 3 M tape mask by the doctor blade method and was heat-treated at 480 °C for 1 hour in air. For measurement, the size of the active area was fixed as 10 mm × 5 mm. N719 dye (0.3 mM; Solaronix) was loaded on the sintered electrode by immersion for 20 h at room temperature. After loading the dye, the textile cells were sealed with 1-mm-thick PET-based laminated pouch film (Sindoh Commerce) using a commercial hot-roll-coating machine (Sindoh Commerce, TL-4600). Acetonitrile-based electrolyte (Solaronix SA, AS50) was injected through a hole that was made by a micro-drill before sealing. After the electrolyte injection, the hole was sealed using a vacuum sealant.

Field-emission scanning electron microscopy (FE-SEM; Hitachi S4800) was performed to observe the surfaces of the samples. The energy-conversion performance of the DSSCs was evaluated using a solar simulator (Abet Technologies, model Sun 2000; 1,000 W Xe source; Keithley 2400 source meter) under 1.5 AM, 1 sun condition, calibrated by a KG-3 filter and NREL-certified reference cell without a mask. The energy-conversion efficiency of the DSSCs under bending and curvature conditions was measured by equipment from Daesung High-tech. The electrochemical characterizations were performed using a BioLogic SP-300 potentiostat. The impedance spectra were acquired under 0 V, 1 sun conditions.

## Additional Information

**How to cite this article**: Yun, M. J. *et al*. Monolithic-Structured Single-Layered Textile-Based Dye-Sensitized Solar Cell. *Sci. Rep.* **6**, 34249; doi: 10.1038/srep34249 (2016).

## Supplementary Material

Supplementary Information
