# Supplementary material for: Monolithic-Structured Single-Layered Textile-Based Dye-Sensitized Solar Cells
Source: Sci Rep. 2016 Oct 6;6:34249. doi: 10.1038/srep34249 (PMC5052609; doi:10.1038/srep34249)
Supplement: Supplementary Information [file srep34249-s1.pdf]

Supplementary Information

Monolithic Structured Single Layered Textile Based Dye-Sensitized Solar Cell

Min Ju Yun[1,2], Seung I. Cha*[1], Han Seong kim[2], Seon Hee Seo[1], , Dong Y. Lee[1]

1. Nano Hybrid Technology Research Center, Creative and Fundamental Research Division, Korea Electrotechnology Research Institute

2. Department of Organic Material Science and Engineering, Pusan National University

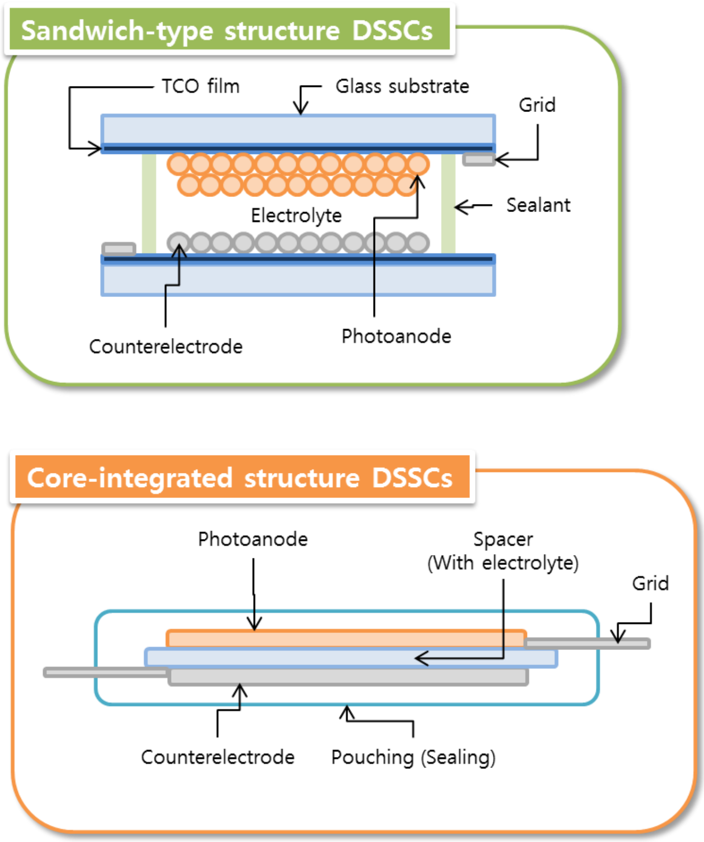

Figure S1. Comparing between the sandwich-type structure DSSC and core-integrated structure DSSC

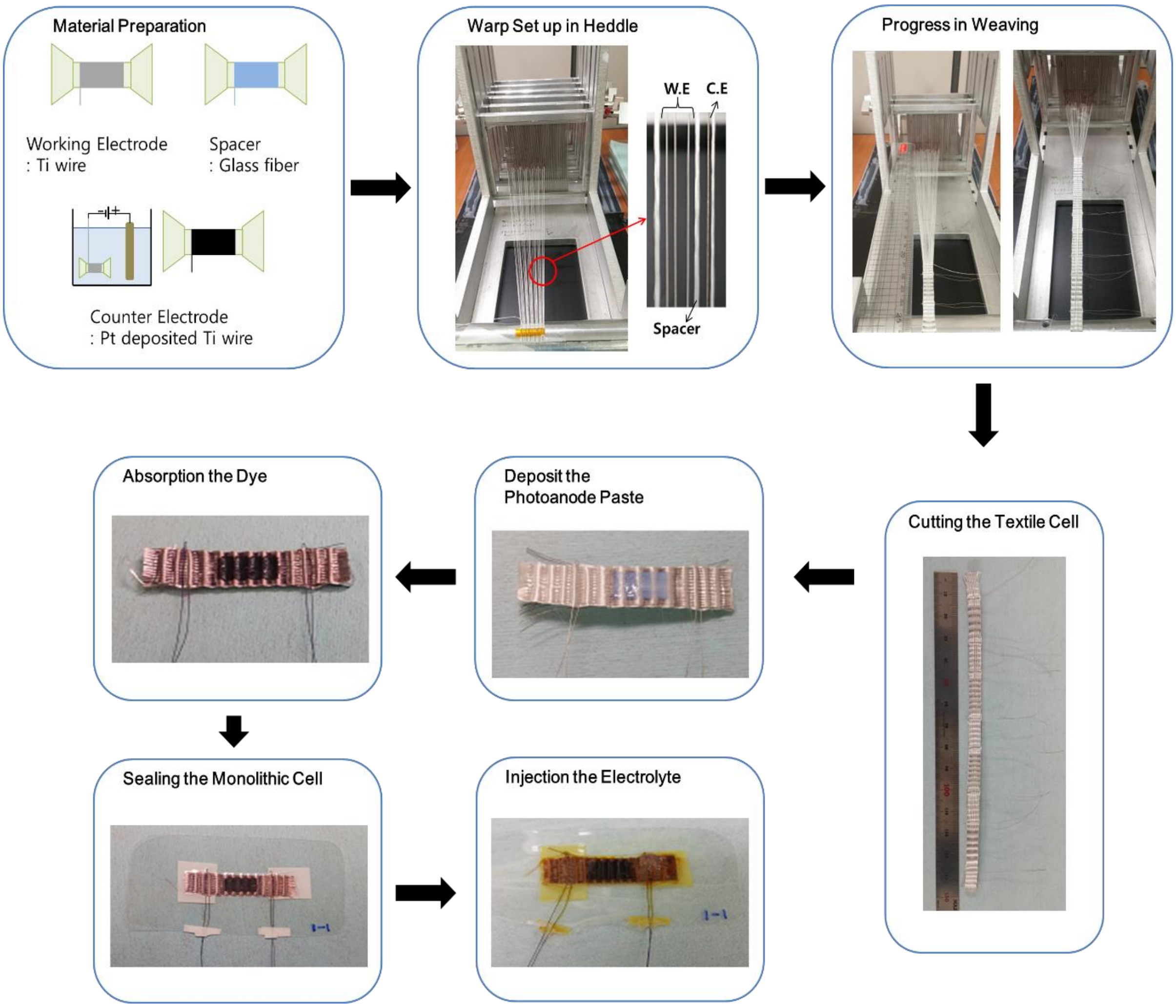

Figure S2. Photographs of all process include setting up the materials, weaving the single layer textile, deposition of the photoanode, stained dye, sealing and injected electrolyte

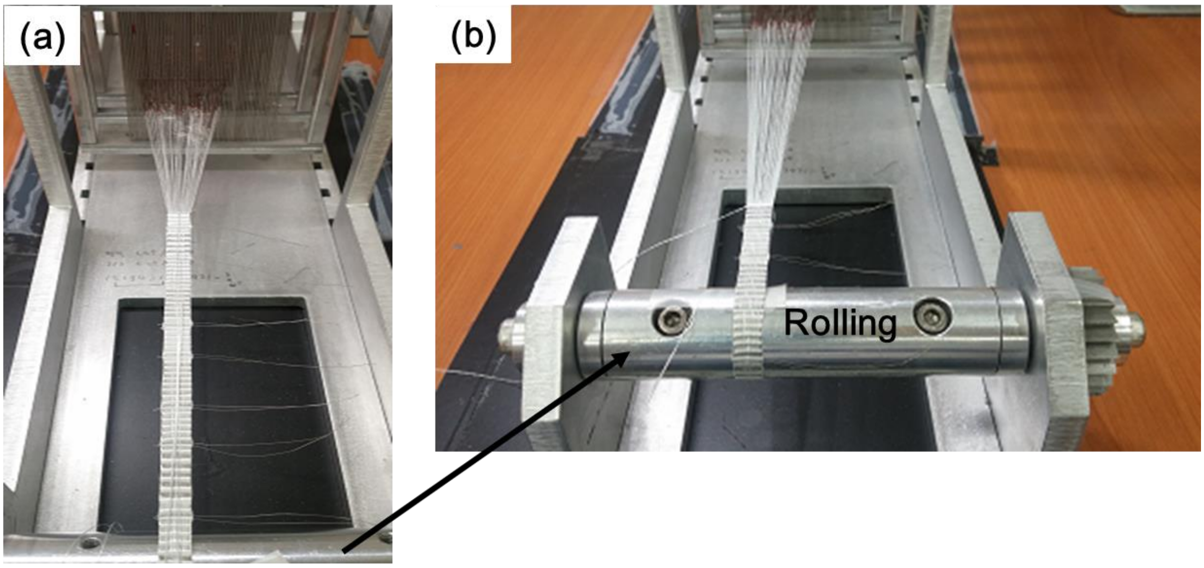

Figure S3. Photograph of (a) roll from the front part of table-top loom and (b) rolled single layer textile cell by rolling

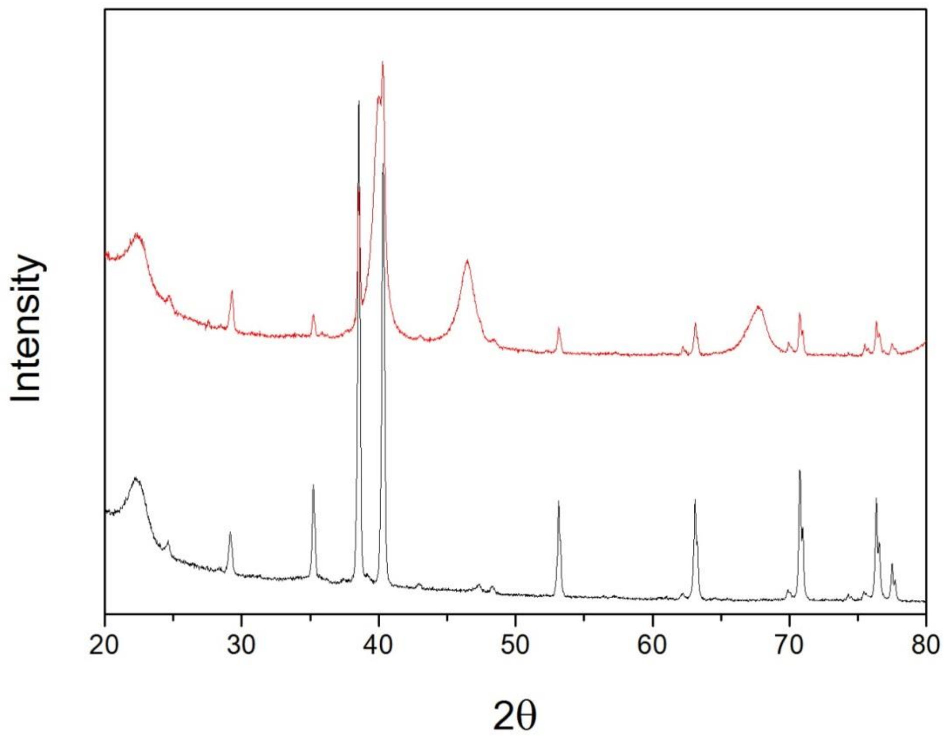

Figure S4. XRD patterns of Ti wire and Pt deposited Ti wire

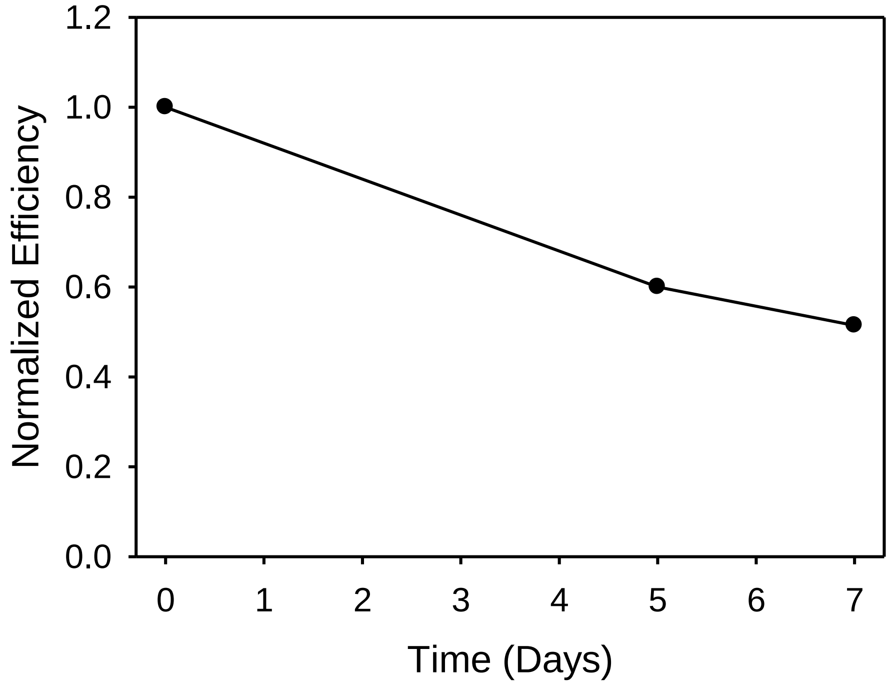

Figure S5. Normalized energy conversion efficiency of 225μm distance between the electrodes with spacing 0.75mm depends on the storage time

Distance between Electrodes (㎛)		Jsc (mA/cm2)	Voc (mV)	Fill Factor	Efficiency (%)
225 (Weft spacing=0.75mm)	Trial ①	1.04	625	0.54	0.35
	Trial ②	0.93	675	0.58	0.36
	Trial ③	0.90	655	0.55	0.32
225 (Weft spacing=1.5mm)	Trial ①	1.42	675	0.65	0.62
	Trial ②	1.32	685	0.63	0.57
	Trial ③	1.36	708	0.60	0.58
350 (Weft spacing=0.75mm)	Trial ①	0.47	542	0.48	0.12
	Trial ②	0.36	550	0.52	0.10
	Trial ③	0.34	585	0.55	0.11
350 (Weft spacing=1.5mm)	Trial ①	1.10	642	0.56	0.40
	Trial ②	0.92	635	0.60	0.35
	Trial ③	0.90	666	0.62	0.37
475 (Weft spacing=0.75mm)	Trial ①	0.60	558	0.41	0.14
	Trial ②	0.78	595	0.35	0.16
	Trial ③	0.66	604	0.31	0.12
475 (Weft spacing=1.5mm)	Trial ①	1.42	610	0.43	0.37
	Trial ②	0.99	644	0.49	0.31
	Trial ③	1.36	615	0.38	0.32

Table S1. Performance of all of trial cells of Monolithic single layer textile DSSC depend on the distance between the electrodes with weft spacing 0.75mm, 1.5mm.
